# Harnessing electronic health record data for improving delivery of acute renal replacement therapy in Alberta, Canada (Dialyzing Wisely)

**DOI:** 10.23889/ijpds.v9i5.2867

**Published:** 2024-09-18

**Authors:** Kristin Robertson, Sheena Morton, Lindsay Dasilva, Tara Whitten, Melissa Gardiner, Sarah Seymour, Dawn Opgenorth, Oleksa Rewa

**Affiliations:** 1 Critical Care Strategic Clinical Network, Alberta Health Services; 2 Provincial Research Data Services, Alberta Health Services; 3 Critical Care and Data Quality/eCritical, Alberta Health Services; 4 University of Calgary; 5 Physician Learning Program; 6 University of Alberta

## Objective

Acute renal replacement therapy (RRT) is an important life saving technology used in critical care. However, variation in clinical practice for initiation and maintenance of RRT can increase healthcare costs and worsen patient outcomes. In Alberta, Canada, a province-wide electronic health record provides a unique opportunity to measure and evaluate clinical practice. The present study aims to improve the quality of RRT delivery in Alberta critical care units by providing feedback on key performance indicators (KPIs) for RRT based on current evidence.

## Approach

KPIs for RRT included an initiation pathway based on threshold lab values, and measures of RRT quality such as the time from order to treatment initiation, the average life of dialysis filters, and the actual vs. prescribed fluid removal. Clinical KPI definitions were mapped to data available in the electronic health record to evaluate current practice and track changes in KPIs over time.

## Results

Mapping the clinical guidelines to the electronic health record took significant time and effort in a large team with expertise on both the data and clinical sides. Each KPI data definition went through several cycles of development, validation and refinement before being included in the dashboards and reports given to participating sites.

## Conclusions

Providing accurate data on RRT ordering and delivery practices is an important step in aligning acute RRT delivery with current best practice, ultimately improving the quality of care and reducing unnecessary costs.

## Implications

Electronic health records provide a powerful tool for evaluating clinical decisions and implementing best practice guidelines.

